# Entomological Efficacy of Aerial Ultra-Low Volume Insecticide Applications Against *Aedes aegypti* (Diptera: Culicidae) in Mexico

**DOI:** 10.1093/jme/tjz066

**Published:** 2019-05-23

**Authors:** Fabián Correa-Morales, Felipe Dzul-Manzanilla, Wilbert Bibiano-Marín, José Vadillo-Sánchez, Anuar Medina-Barreiro, Abdiel Martin-Park, Josué Villegas-Chim, Armando E Elizondo-Quiroga, Audrey Lenhart, Gonzalo M Vazquez-Prokopec, José Erales-Villamil, Azael Che-Mendoza, Pablo Manrique-Saide

**Affiliations:** 1 Centro Nacional de Programas Preventivos y Control de Enfermedades (CENAPRECE) Secretaría de Salud Mexico, CDMX, Mexico; 2 Collaborative Unit for Entomological Bioassays, Campus de Ciencias Biológicas y Agropecuarias, Universidad Autónoma de Yucatan. Merida, Yucatan, Mexico; 3 Secretaria de Salud Jalisco, Guadalajara, Jalisco, Mexico; 4 Centers for Disease Control and Prevention, Center for Global Health/Division of Parasitic Diseases and Malaria/Entomology Branch, Atlanta, GA; 5 Department of Environmental Studies, Emory University, Atlanta, GA

**Keywords:** vector control, ultra-low volume, aerial application, RCT, Mexico

## Abstract

A cluster-randomized controlled trial quantified the entomological efficacy of aerial ultra-low volume (AULV) applications of the insecticide chlorpyrifos against *Aedes aegypti* in Puerto Vallarta, México, during November–October 2017. The trial involved 16 large (1 × 1 km) clusters distributed between treatment-control arms. Primary endpoint was the abundance of *Ae. aegypti* indoors (total adults, females, and blood-fed females) collected using Prokopack aspirators. After four consecutive weekly cycles of AULV, all adult *Ae. aegypti* infestation indices were significantly lower in the treatment arm (OR and IRR ≤ 0.28). Efficacy in reducing indoor *Ae. aegypti* increased with each weekly application cycle from 30 to 73% (total adults), 33 to 76% (females), and 45.5 to 89% (blood-fed females). Entomological indices remained significantly lower in the treatment arm up to 2 wk after the fourth spraying round. Performing AULV spraying can have significant and lasting entomological impact on *Ae. aegypti* as long as multiple (ideally four) spray cycles are implemented using an effective insecticide.

Over the past three decades, urban mosquito-borne virus epidemics have increased in magnitude and frequency (Gubler 2011, Rosenberg et al. 2018). These epidemics require rapid and widespread case management and vector control actions (Vazquez-Prokopec et al. 2010, Redd and Frieden 2017). Due to the typically reactive nature of urban vector control, most vector control activities have been unsuccessful at rapidly containing outbreaks due to lack of trained staff and insufficient resources, as well as political barriers to the rapid allocation of resources (Morrison et al. 2008). Aerial ultra-low volume (AULV) spraying of insecticides is a recommended practice as part of the Integrated Vector Management (IVM) program if its included in a proactive way, and a method for the rapid control of adult mosquito populations during outbreaks over large urban areas (WHO 2003, Bonds 2012, CDC 2013, Ruktanonchai et al. 2014), especially where access with ground equipment is difficult and extensive areas (>1,000 ha) need to be treated rapidly. AULV involves the application with a low-flying aircraft of an adulticide as a cold aerosol with a droplet size ranging between 25 and 40 μm (Bonds 2012). In the United States, this method is commonly employed to reduce mosquito populations as part of a proactive Integrated Mosquito Management (IMM) program (CDC 2013, 2018). It has also been applied on a limited scale for vector-borne disease control, with evidence suggesting that the successful containment of West Nile Virus outbreaks in California and Texas was due to AULV spraying (Carney et al. 2008, Ruktanonchai et al. 2014).

AULV has been historically recommended for the emergency control of dengue outbreaks in the Americas (OPS 1981), although it has not been routinely used in most dengue-endemic countries. Most recently, AULV was employed as part of an IVM response to the Zika outbreak in Florida to control urban populations of *Aedes aegypti* (Likos 2016, CDC 2018). In the Florida context, it is unclear if the successful containment of the Zika outbreak was due to AULV or its use in combination with other measures (ground ULV spraying of larvicides, source reduction campaigns, and community education). Discrepancies exist regarding the efficacy of ultra-low volume (ULV) spraying (both aerial and ground-level applications) in controlling *Ae. aegypti* populations (Reiter and Nathan 2001, McCall 2006, Reiter 2014), primarily due to lack of data from well-designed and executed studies evaluating entomological and epidemiological endpoints (Bowman et al. 2016). To better define the role that AULV could have in urban *Ae. aegypti* control, we conducted a cluster-randomized controlled trial to evaluate the entomological efficacy of AULV spraying to control adult *Ae. aegypti* following WHO standard protocols (WHO 2003) and testing requirements established in Mexico.

## Materials and Methods

### Study Design

**Fig. 1.**
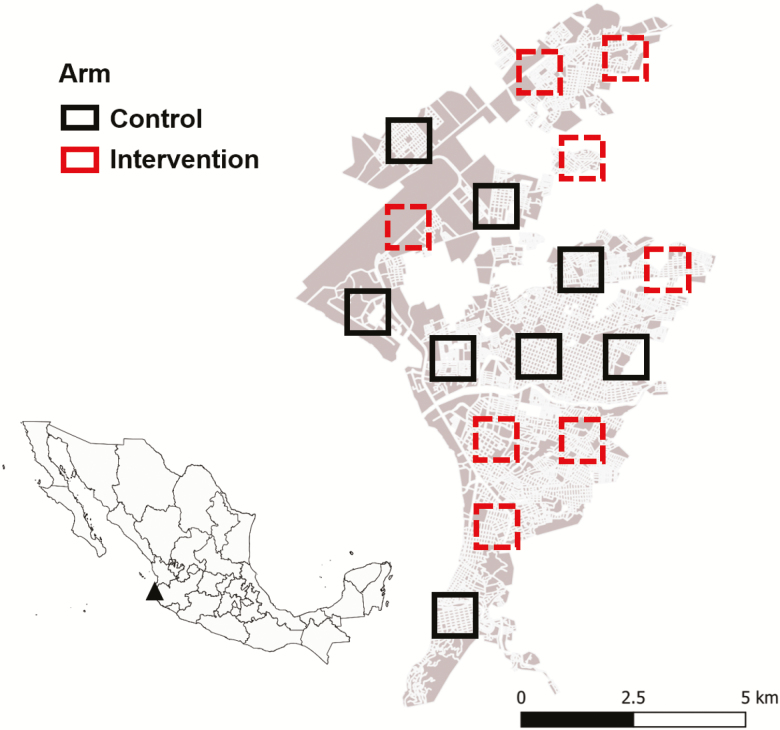
Distribution of clusters (1 km^2^) on each arm of the trial. Inset shows the location of Puerto Vallarta in Mexico.

The trial was performed in Puerto Vallarta, Mexico (Fig. 1; 20°40′N 105°16′O/20.667, −105.267) comprising of 1,300 km^2^ and 255,681 inhabitants located in the northern coastal region of the Mexican state of Jalisco on the Pacific coast. The climate is tropical and subhumid, with average high temperatures ranging from 20 to 32°C and with an average annual rainfall of 1,100–2,000 mm occurring from June to November (GPV 2015, INEGI 2018). The rainy season coincides with the peak of *Ae. aegypti* abundance and the period of highest transmission of *Aedes*-borne viruses (Jalisco Ministry of Health, unpublished data).

This study was a two-arm cluster-randomized controlled trial (CRCT) performed in 16 clusters of 1 km^2^ (100 ha) with at least 250 houses per km^2^ (Fig. 1). The study was designed to simulate an emergency response of the Mexican Ministry of Health (MoH). The study was conducted during the peak season of *Ae. aegypti* abundance (August–November) in 2017. Treatments followed a 1:1 allocation, with eight clusters randomly assigned to intervention (AULV spraying) and eight clusters for control (no AULV spraying; Fig. 1). Routine MoH vector control interventions (adulticiding—e.g., outdoor and indoor spraying with chlorpyrifos and bendiocarb, respectively—larviciding—e.g., Spinosad-, and anti-*Aedes* health education measures in response to the notification of clinical dengue cases) occurred throughout the city irrespective of cluster allocation. The clusters were delineated in accordance with the local MoH to include areas of epidemiological importance for the local dengue control program. A minimum 1-km buffer distance separating clusters was used to minimize contamination in estimates of efficacy.

### AULV Application

Given the high levels of pyrethroid resistance in *Ae. aegypti* in Mexico (Kuri-Morales et al. 2018), the organophosphate MOSQUITOCIDA UNO ULV (Active ingredients: Chlorpyrifos [0,0-diethyl-0-(3,5,6,-trichloro-2-pyridil) phosporothioate] 13.624%, Oil Soluble, Public Health Supply and Equipment of Mexico, Mexico) was used. This insecticide is approved by Mexican regulatory authorities for use as a mosquito adulticide in open spaces and as ULV for public health control of mosquitoes (CENAPRECE 2017). The insecticide was applied at 27.2 gr. a.i. of chlorpyrifos/ha, following the Mexican MoH recommendations.

**Fig. 2.**
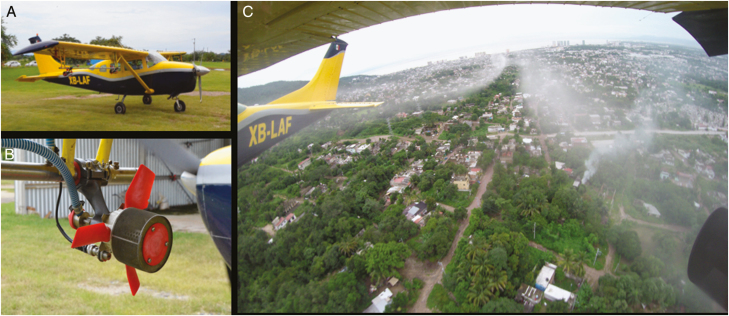
(A) Aircraft employed in the trials with (B) the rotary atomizer mounted under wing and (C) aerial view during the application.

Four cycles of 1-d single spray treatments (i.e., one spray every week for four consecutive weeks) in each intervention cluster were performed from September 27th to October 18th of 2017. Briefly, a Mexico MoH-certified company (Aerofumigaciones del Valle) was contracted to perform AULV applications for the trial using a Cessna 206 aircraft equipped with Flightmaster drift optimization software (AG-NAV Flightmaster, Ontario, Canada) and four Micronair AU 4000 (Goizper-Micron Group, Bromyard, United Kingdom) rotary atomizers (two atomizers mounted under each wing; Fig. 2). The spraying equipment was calibrated to provide a droplet size of 25–45 μm, a blade speed of 7,800 rpm, and a flow rate of 9,390 ml/min. The Cessna 206 flew at an altitude of 60 m with a swath width of 146 m and speed of 193 km/h (120 mph). Applications were conducted at dawn (7:45 a.m.) during favorable environmental conditions (e.g., no rain or winds higher than 16 km/h).

### Entomological Evaluation

We performed a baseline entomological survey 1 wk prior the intervention to quantify indoor *Ae. aegypti* abundance using Prokopack aspirators (Emory University, Atlanta; Vazquez-Prokopec et al. 2009), collecting for 10 min per house, in a random sample of 30 houses per cluster (480 houses in total). The same methodology was performed weekly after initiation of AULV applications for a total of six follow-up weeks, consisting of four collections 24 h after each AULV application cycle and two collections the 2 wk that followed the last AULV application.

### Statistical Analyses

Household-specific entomological indices derived from indoor adult mosquito collections (i.e., presence and number of total adults, females, and blood-fed *Ae. aegypti*) were analyzed with generalized linear mixed models (GLMM) as in Vazquez-Prokopec et al. (2017). For presence/absence indices, binomial GLMMs were used, using arm as a fixed effect and cluster as the random intercept. For abundance indicators, negative-binomial GLMMs were used, also with arm as a fixed effect and cluster as the random intercept. The logistic regression coefficients were transformed to odds ratios (OR) and the exponentiated negative binomial coefficients to incidence rate ratios (IRR), to facilitate interpretation (Vazquez-Prokopec et al. 2017). Confidence intervals (95% CI) were calculated using bootstrap methods. An OR or IRR significantly lower than 1 (based on their 95% confidence intervals) indicated a significant reduction (*P* < 0.05) for a given entomologic metric. Finally, we calculated the efficacy of the intervention as *E = (1-IRR)* * 100 (Vazquez-Prokopec et al. 2017). *E* describes the percent reduction of mosquito abundance in treated houses with respect controls. R software packages lme4 (Bates et al. 2015), broom (Robinson 2017), tidyverse (Wickham 2017), and purrr (Henry and Wickham 2017) were used.

### Ethics Statement

The project entitled “Evaluating the Impact of Aerial Ultra- Low Volume (AULV) Spraying for Zika Vector Control Services” was reviewed and approved by the institutional ethics committee of the Ministry of Health of Yucatan. Products and methods applied are already in use in Mexico and are in agreement to the current ethical normativity and are recommended by the Ministry of Health of Mexico.

## Results

**Fig. 3.**
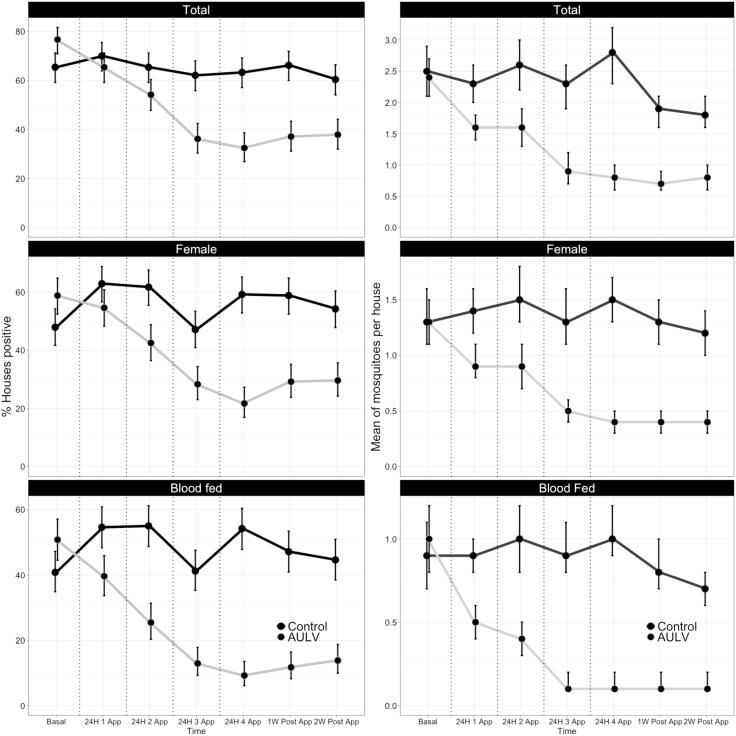
Entomological metrics of indoor *Ae. aegypti* infestation (% houses positive, left column) and abundance (average number collected per house, right column) calculated for intervention (gray lines) and control (black lines) arms before, during, and after AULV application in Puerto Vallarta, Jalisco, México. Vertical dotted lines show each of the four AULV applications.

At baseline, the prevalence of house infestation (% of positive houses) measured for the three *Ae. aegypti* indices (adults, females, and blood-fed females) was ~10% higher in the treatment compared with the control arm (Fig. 3) and was statistically significant for all indices (Table 1). House infestation indices in control clusters remained high throughout the trial, ranging from 60 to 70% for total adults, 48 to 62% for females, and 40 to 52% for blood-fed females (Fig. 3). Immediately (24 h) after the first AULV application, there was a slight decrease in house infestation indices in the AULV arm (Fig. 3), which was nonsignificant for total adults and females but statistically significant for blood-fed females (Table 1). A linear and significant reduction in house infestation indices was observed after the second round of AULV spraying and continued after each subsequent spray event, with all three indices remaining significantly lower in the treatment arm compared with the control arm up to 2 wk after the final (fourth) AULV application (Fig. 3, Table 1). Interestingly, the slight increase in house infestation indices in the treatment arm 2 wk after the final AULV spraying was not statistically different from the values that were detected 24 h after the final AULV treatment (Fig. 3), suggesting a lasting suppression of *Ae. aegypti* prevalence.

**Table 1.** OR and IRR of three *Ae. aegypti* adult abundance indices, calculated from mixed-effects logistic and negative binomial regression models evaluating the impact of AULV spraying on each adult entomological outcome in Puerto Vallarta, Jalisco, México

Outcome	Survey	IRR		OR	
		Coefficient (Lower–Upper)	*P*	Coefficient (Lower–Upper)	*P*
Total *Ae. aegypti* (males and females)	Baseline	1.74 (1.15–2.63)	0.01	0.97 (0.74–1.27)	0.81
	After first application (24 h)	0.81 (0.51–1.28)	0.37	0.70 (0.53–0.91)	0.01
	After second application	0.62 (0.41–0.94)	0.3	0.59 (0.41–0.84)	0.00
	After third application	0.34 (0.21–0.55)	0.00	0.41 (0.27–0.60)	0.00
	Fourth application	0.28 (0.19–0.41)	0.00	0.27 (0.19–0.39)	0.00
	1 Wk postapplication	0.28 (0.15–0.53)	0.00	0.39 (0.27–0.57)	0.00
	2 Wk postapplication	0.40 (0.27–0.58)	0.00	0.43 (0.34–0.56)	0.00
*Ae. aegypti* females	Baseline	1.55 (1.08–2.22)	0.02	1.01 (0.70–1.46)	0.95
	After first application (24 h)	0.71 (0.48–1.04)	0.08	0.67 (0.52–0.86)	0.00
	After second application	0.45 (0.28–0.74)	0.00	0.53 (0.37–0.77)	0.00
	After third application	0.44 (0.28–0.70)	0.00	0.39 (0.25–0.34)	0.00
	Fourth application	0.19 (0.13–0.28)	0.00	0.24 (0.17–0.47)	0.00
	1 Wk postapplication	0.28 (0.16–0.48)	0.00	0.32 (0.21–0.48)	0.00
	2 Wk postapplication	0.36 (0.24–0.52)	0.00	0.36 (0.27–0.5)	0.00
*Ae. aegypti* blood-fed females	Baseline	1.50 (1.04–2.15)	0.03	1.11 (0.76–1.64)	0.59
	After first application (24 h)	0.55 (0.38–0.78)	0.00	0.55 (0.42–0.70)	0.00
	After second application	0.27 (0.16–0.46)	0.00	0.37 (0.23–0.59)	0.00
	After third application	0.21 (0.13–0.34)	0.00	0.16 (0.09–0.27)	0.00
	Fourth application	0.09 (0.05–0.14)	0.00	0.11 (0.07–0.18)	0.00
	1 Wk postapplication	0.14 (0.08–0.26)	0.00	0.15 (0.09–0.24)	0.00
	2 Wk postapplication	0.19 (0.10–0.34)	0.00	0.19 (0.11–0.32)	0.00

For all comparisons, OR and IRRs are calculated using control arm as the comparator.

Contrary to the house infestation indices, adult abundance indices were not statistically different between treatment and control arms at baseline (Fig. 3, Table 1). Control clusters had an average abundance ranging from 1.8 to 2.8 adults per house, 1.2 to 1.5 females per house, and 0.7 to 1.0 blood-fed females per house (Fig. 3). AULV applications significantly reduced *Ae. aegypti* abundance in the treatment arm (Fig. 3), with all indices being significantly lower than in the control arm after the first and all subsequent AULV applications (Table 1). As with the house infestation indices, a linear reduction in all adult abundance indices was observed after each round of AULV, leading to sustained effects up to 2 wk after the fourth spraying event (Fig. 3, Table 1).

**Fig. 4.**
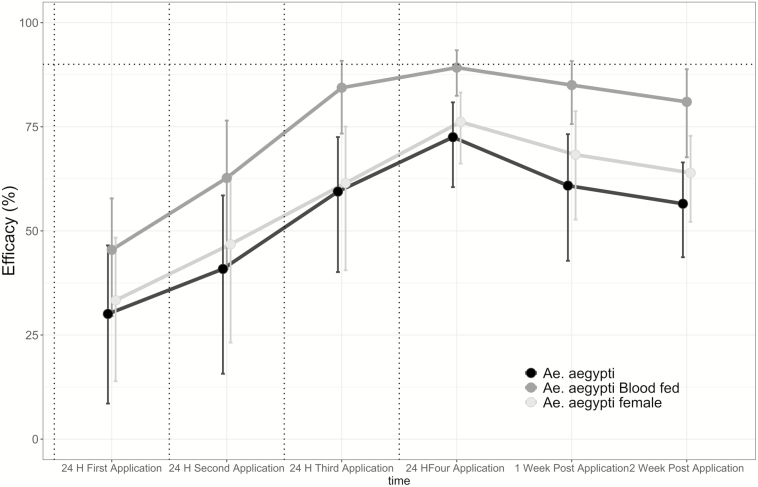
Efficacy of AULV application of the organophosphate chloropyrifos on *Ae. aegypti* adult indices indoors in Puerto Vallarta, Jalisco, Mexico. The vertical dotted lines show each of the four AULV applications and the horizontal line represents the reduction threshold of 90% (the indoor threshold for efficacious AULV applications recommended by Mexican Official Standards, DOF 2015).

Using the values from the negative-binomial GLMM models, we estimated the average efficacy (*E*) of AULV in reducing the abundance of *Ae. aegypti* indoors (Fig. 4). Efficacy of AULV in the reduction of adult abundance after the four application cycles increased from 30% after the first spray event to 72.5% after the fourth spray event (Fig. 4; E_1st_ = 30.1, E_2nd_ = 40.9, E_3rd_ = 59.4, E_4th_ = 72.5). A similar trend in increase of efficacy with subsequent AULV applications was observed for the other adult indices, with *E* increasing from 33 to 76% for females (E_1st_ = 33.3, E_2nd_ = 46.8, E_3rd_ = 61.5, E_4th_ = 76.2) and from 45.5 to 89% for blood-fed females (E_1st_ = 45.5, E_2nd_ = 62.7, E_3rd_ = 84.4, E_4th_ = 89.2; Fig. 4). Efficacy decreased after the fourth AULV application, but was higher than 50% up to 2 wk after the final spray event (E_2Weeks post_ =55.9 for adults, E_2Weeks post_ = 63.9 for females, and E_2Weeks post_ = 81 for blood-fed females; Fig. 4).

## Discussion

The efficacy of AULV spraying in controlling adult *Ae. aegypti* populations has been under debate in recent years, primarily arising from the findings of studies conducted in the Americas in the 1980s and 1990s (Fox 1980, Perich et al. 1990, Castle et al. 1999). These previous experiences with AULV demonstrated limited entomological impact, primarily after single AULV applications. For example, Fox (1980) found a lack of efficacy after a single AULV application with malathion for following 3 mo on the infestation levels of larvae and pupae within cement vases in a cemetery in San Juan, Puerto Rico. Similarly, Castle et al. (1999) concluded that a single application of AULV with malathion did not show significant effects on *Aedes* egg counts from ovitraps in Kingston, Jamaica. Perich et al. (1990) also reported a lack of post‐treatment impact on egg counts from ovitraps or indoor adult collections with sweep nets in Santo Domingo, Dominican Republic following two applications (within 5 d) AULV spraying with malathion. However, important reductions in *Ae. aegypti* abundance after repeated AULV applications of naled (dimethyl 1,2-dibromo-2,2-dichloroethylphosphate) in combination with turbine-dispersed Bti were observed in Miami-Dade after the introduction of Zika virus in 2016 (Likos et al. 2016). Our study, which followed a randomized cluster design, found initial levels of efficacy after a single AULV application of 27% for adults, 28% for females, and 45% for blood-fed females, but improved efficacy with subsequent applications. When an insecticide to which *Ae. aegypti* is susceptible is used, weekly AULV applications acted in an additive fashion, further suppressing populations with each repeated weekly application; high (>70%) levels of control were maintained for up to 2 wk after a four-cycle weekly application. Our study supports early findings indicating that a single AULV application is not enough to reach large population-level impact on *Ae. aegypti* and point to the need of repeated weekly applications in order to achieve significant entomological impacts. A similar conclusion with regard to the need of repeated spraying cycles has been reported for ground ULV for vector control (Likos et al. 2016, Chaskopoulou et al. 2018).

One of the key arguments against using AULV spraying to control adult *Ae. aegypti* is that the spray droplets do not reach mosquitoes resting indoors. Britch et al. (2018) recently performed an experimental study investigating the capability of the organophosphate naled (Dibrom) applied from a fixed wing ULV spray platform to penetrate indoor habitats containing adult *Ae. aegypti* mosquitoes. They found that the aerial applications of naled varied in their ability to penetrate indoors and reported mortalities of 68.9% in mosquitoes within sentinel cages placed indoors.

In this publication, the authors also included entomological efficacy results after AULV spraying with naled from other authors, which ranged from 71.0 to 75.3% as assessed based on mortality of caged *Ae. aegypti* (Merk et al. 1971, CDC 1987). Our study supplements such findings and shows that, despite being applied outdoors, AULV can have a significant impact on free-flying indoor resting *Ae. aegypti*. However, in cities with more enclosed housing structures or a great number of high buildings (which limit the range of low-flying aircraft), the results may not necessarily be the same. As such, the choice of adulticiding, and specifically AULV, should be done based on knowledge of the local *Ae. aegypti* population, the housing characteristics of the city, and community acceptance for this method.

Our study did not collect information about insecticide movement and its deposition that could help explain our findings. We hypothesize that AULV, applied sequentially, was successful on reducing outdoor adult populations of *Ae. aegypti*, and consequently, reduced significantly the indoor recruitment of adults entering the home environment. This can be considered an alternative explanation to the proposed by Britch and cols (2018), who establishes that the insecticidal effect of a formulation applied as ULV could also act as a vapor, and not only as droplets. We discarded such hypothesis in our study because the insecticide used was an oil-based formulation, more resistant to evaporation compared with water-based formulation. Since our study relied on a well-powered cluster randomized design and was carried out using state-of-the-art technology (drift optimization software) to improve the accuracy and precision of insecticide application (e.g., using computer modeling that offset spray lines to account for pattern displacement and potential drift caused by the wind or other weather conditions), we consider our findings to be a reflection of the true effect of AULV applications on natural *Ae. aegypti* populations.

Insecticide resistance is another challenge faced by any insecticide-based interventions. Earlier studies on AULV failed to provide data regarding levels of insecticide resistance to the insecticide(s) applied in the local *Ae. aegypti* populations. Prior to spraying, we confirmed that *Ae. aegypti* was susceptible to the organophosphate chlorpyrifos (Kuri-Morales et al. 2018). As such, we hypothesize that the low initial efficacy was due to shortcomings inherent in the AULV application (failure to reach all mosquitoes indoors, time-lags between insecticide application and reduction in indoor adult abundance, high canopy cover, etc.) and not to insecticide resistance in the local mosquito population. Given the strong negative effect that insecticide resistance can have on the efficacy of interventions (Vazquez-Prokopec et al. 2017), it is crucial that AULV and any other interventions be implemented within an insecticide resistance management plan. For instance, Mexico’s MoH regulates the use of insecticides, and regulations are supervised and adapted over time and in response to insecticide resistance trends (CENAPRECE 2015). The successful emergency application of AULV during outbreaks would require mechanisms for insecticide procurement that are both prompt and based on the resistance profile of the local mosquito populations.

During epidemics, rapid control of *Ae. aegypti* over large urban areas can theoretically be carried out with AULV spraying, especially in settings where access with ground equipment is difficult and extensive areas need to be treated very rapidly (Gratz 1991, WHO 2003, Bonds 2012). Although AULV is one of the methods currently available for outbreak control, its implementation in the context of *Ae. aegypti* control in endemic developing countries is rare (McCall 2006). Indeed, AULV spraying is not recommended as a routine preventive method for *Aedes*-borne viruses because lack of information on its epidemiological impact and limited information about cost-effectiveness of implementation (Achee et al. 2015). Part of the debate challenging AULV is the assumption of a short-lived entomological impact (it was assumed that adult mosquito populations would return to pretreatment levels within 2 wk, Newton and Reiter 1992). The finding of sustained reductions in *Ae. aegypti* abundance even 2 wk after the fourth AULV round provide important new evidence about the duration of the entomological impact of this intervention. Perhaps, if rapidly implemented once an outbreak is declared, multiple cycles of AULV can lead to a significant impact on virus transmission at the city level. It remains to be studied if large-scale control with AULV, using insecticides to which *Ae. aegypti* are susceptible, can potentially impact virus transmission in a meaningful way. Performing randomized controlled trials with epidemiological endpoints would be a next step in the evaluation of this methodology.
